# Early Detection of Palliative Care Needs in Critically Ill Patients Using the NECPAL Tool

**DOI:** 10.3390/jcm14176244

**Published:** 2025-09-04

**Authors:** Savino Spadaro, Danila Azzolina, Adelaide Vuan, Luigi Colasanto, Cristiana Manzetto, Alessandra Busnardo, Grazia Filieri, Persefoni Fagogeni, Francesco Ganzaroli, Viorel Gamanji, Giovanni Poles, Giorgia Spinazzola, Carlo Alberto Volta, Gaetano Scaramuzzo, Loretta Gulmini

**Affiliations:** 1 Emergency Department, Azienda Ospedaliera Universitaria di Ferrara, Aldo Moro, 8, 441232 Ferrara, Italy; savino.spadaro@unife.it (S.S.); vlc@unife.it (C.A.V.); scrgtn@unife.it (G.S.); 2 Department of Translational Medicine, University of Ferrara, 44124 Ferrara, Italy; adelaide.vuan@edu.unife.it (A.V.); l13.colasanto@gmail.com (L.C.); cristiana.manzetto@gmail.com (C.M.); grazia.filieri@unife.it (G.F.); persefoni.fagogeni@gmail.com (P.F.); francesco.ganzaroli@unife.it (F.G.);; 3 Biostatistics and Clinical Trial Methodology Unit, Clinical Research Center DEMeTra, Department of Translational Medicine, University of Naples Federico II, 80138 Naples, Italy; 4 Unità Complessa Cure Palliative AULSS3 Serenissima, Venezia Mestre, Cappuccina, 129, 30172 Venezia, Italy; giovanni.poles@aulss3.veneto.it; 5 Department of Anesthesia and Intensive Care, Fondazione Policlinico Universitario A. Gemelli IRCCS, 00136 Rome, Italy; giorgia.spinazzola@policlinicogemelli.it; 6 Cure Palliative/Hospice, Azienda Ospedaliera Universitaria di Ferrara, Cavallotti 347, 44100 Ferrara, Italy; gulminiloretta@gmail.com

**Keywords:** palliative care, intensive care setting, chronic disease, advanced frailty, quality of life

## Abstract

**Background**: Palliative care is essential in intensive care, improving symptom control, quality of life, and reducing hospital stays without increasing mortality. However, early identification of patients who could benefit remains a major challenge. This study aimed to evaluate the NECesidades PALiativas (NECPAL) tool’s effectiveness in identifying ICU patients in Italy with PC needs. **Methods**: This prospective observational study was conducted from March 2024 to February 2025. Adult ICU patients (≥18 years), admitted for at least 24 h and meeting NECPAL eligibility criteria (e.g., cancer, COPD, heart failure, dementia, and frailty), were evaluated using the NECPAL tool. **Results**: A total of 85 patients were enrolled; 28 (32.9%) were classified as NECPAL-positive and 57 (67.1%) as NECPAL-negative. NECPAL-positive patients had a significantly higher ICU mortality rate (32.1%) compared to NECPAL-negative patients (1.8%) (*p* < 0.001). The median ICU length of stay was slightly longer for NECPAL-positive patients [11.0 days (IQR: 8.0–16.2)] versus NECPAL-negative patients [10.0 days (IQR: 5.0–14.0)], though not statistically significant. Multivariable Firth penalized logistic regression confirmed NECPAL positivity as an independent predictor of ICU mortality (OR 19.55; 95% CI: 3.06–124.93; *p* < 0.001). **Conclusions**: In this study, NECPAL identified about one-third of ICU patients as having palliative care needs, who also showed a higher mortality risk. Integration with ICU severity scores may improve early recognition of these needs, warranting validation in larger multicenter studies.

## 1. Introduction

Palliative care (PC) has progressively evolved to address a broad spectrum of illnesses, with a particular emphasis on progressive, chronic, non-cancer conditions [1].

Over recent decades, demographic and epidemiological transitions have contributed to increased life expectancy, thereby leading to an aging population and a growing burden of chronic, progressive illnesses. As a result, the demand for PC has risen significantly [2]. Nonetheless, despite this increasing need, individuals with chronic conditions frequently experience delayed and limited access to PC services [3], leading to a higher incidence of emergency department visits and intensive care unit admissions [4].

In the ICU setting, where patients often face complex illness trajectories and high mortality risk, the role of PC is particularly crucial. PC, considering its aim of relieving pain, symptoms, and stress caused by serious illness, is considered essential in intensive care units (ICUs), despite ongoing high mortality rates and increasing intensive treatments at the end of life [5,6]. Recent evidence highlights the advantages of early integration of patient-centered PC in the ICU, demonstrating its role in preventing overtreatment, enhancing symptom control and quality of life, shortening ICU length of stay without impacting mortality, and alleviating stress for both families and healthcare providers [7,8,9,10].

Despite the advantages, there are several challenges to implementing PC in the ICU. One significant barrier is the difficulty in identifying patients who could benefit from PC at an early stage [11]. Prognostic accuracy may be insufficient [12], and ICU clinicians often fail to consider the patient’s post-hospitalization trajectory [13]. Furthermore, PC needs are frequently underrecognized in critical care settings [14]. Accordingly, the prompt identification of patients with PC needs and the implementation of validated assessment tools are essential to ensure appropriate and timely care delivery.

The NECPAL tool [15,16] is a valid and effective screening instrument for identifying patients who require PC [16,17]. This tool, which uses clinical parameters and the Surprise Question (SQ), is designed to detect patients with advanced chronic diseases and limited life expectancy who may benefit from PC [15,16]. NECPAL goes beyond mortality prediction by integrating clinical, functional, and multidimensional indicators, offering a more comprehensive screening tool than approaches like the SQ [17].

Despite the growing emphasis on early PC, there is currently a lack of evidence regarding the use of the NECPAL tool to assess PC needs specifically within the critically ill population. In intensive care settings, the NECPAL tool serves to identify patients who could benefit from different levels of PC, including early integration, concurrent support alongside curative treatments, and specialized palliative interventions [9]. PC implementation does not replace ongoing treatments but complements them, often improving care by tailoring interventions to the individual needs of the patient. This structured approach may help clinicians balance aggressive therapies with patient-centered goals, ensuring interventions are tailored to individual needs and values.

Given the complexity of clinical decisions and challenges in identifying patients needing PC, the NECPAL tool provides a systematic method to facilitate early detection. Furthermore, formalizing the recognition of palliative needs, NECPAL can prompt timely discussions with patients and families, reducing conflict and improving shared decision-making.

Based on this, we hypothesize that the use of the NECPAL tool facilitates earlier and more appropriate integration of PC services in critical care.

The primary aim of this study is to evaluate the usefulness of the NECPAL tool in identifying PC needs among adult patients admitted to the ICU.

## 2. Materials and Methods

### 2.1. Study Design

This prospective observational study was conducted from March 2024 to February 2025 in the Intensive Care Unit (ICU) of the Azienda Ospedaliero-Universitaria di Ferrara, Italy.

### 2.2. Participants

The inclusion criteria were (1) adult patients aged 18 years or older admitted to the Intensive Care Unit for at least 24 h, and (2) eligibility for NECPAL assessment based on the presence of advanced medical conditions such as cancer, chronic obstructive pulmonary disease, chronic heart disease, chronic neurological disorders, severe chronic liver or renal disease, dementia, advanced frailty, or other similarly severe chronic illnesses. Patients who were already receiving PC services were excluded.

### 2.3. Study Outcomes

The primary outcome was the NECPAL tool’s usefulness in predicting ICU mortality. Secondary outcomes assessed its ability to predict ICU length of stay, alongside evaluating the SQ’s performance in predicting both mortality and ICU length of stay related to PC needs.

### 2.4. Variables and Tools

The NECPAL CCOMS-ICO© V.1.0 [18] was administered within 48 h of ICU admission.

This tool has been widely utilized in various research settings [15,19]. It comprises four items, the first of which is the SQ (SQ, Item 1): ‘Would you be surprised if this patient died within the next year?’.

The other 3 items explore, respectively, (Item 2) the choice/request or need for a PC approach (I2); (Item 3) the general clinical indicators of severity and progression, including comorbidity and resource use (I3); (Item 4) and specific clinical indicators of severity and progression for various diseases (I4). Patients in which the NECPAL questionnaire was not possible, for lack of information or documentation, were excluded from the analysis. Based on the questionnaire results, patients were categorized as NECPAL+ or NECPAL−. A positive NECPAL identification (NECPAL+) was assigned to patients with a positive response to the SQ (‘No, I would not be surprised if this patient died within the next 12 months’) along with at least one additional positive item from the remaining three components of the tool [20]. For all included patients, the following data were collected, as follows: age, sex, comorbidities, number of hospital admissions in the preceding 12 months, pre-admission Karnofsky Performance Status [21], and Clinical Frailty Scale scores [22,23]. Additional information included the source of admission to the ICU (e.g., another ICU, operating room, or stepdown unit), length of hospital stay prior to ICU admission, admission diagnosis, need for ventilatory support upon ICU admission, ICU length of stay, and ICU outcome.

### 2.5. Ethics Approval and Consent to Participate

The study was approved by the Ethics Committee at Azienda Ospedaliera of Ferrara (ID (024/2023/Oss/AOUFe). All participants provided written informed consent prior to participation.

### 2.6. Statistical Analysis

Categorical variables were described using absolute frequencies (n) and percentages (%), whereas continuous variables were summarized as medians with interquartile ranges (IQRs). All data summaries were conducted for the overall sample and stratified by group as appropriate. Group comparisons assessed differences in baseline characteristics and outcomes between cohorts. Continuous variables were compared using the Mann–Whitney U test; categorical variables were compared using Pearson’s Chi-square test or Fisher’s exact test when expected cell counts were <5 in any category. The results of these comparisons are reported with corresponding *p*-values for the difference between groups. All hypothesis tests were two-tailed.

Multivariable regression models were fitted for binary, intrahospital mortality, and continuous endpoint, length of stay (LoS), to identify independent predictors of ICU outcomes. Two separate models were estimated, including (1) NECPAL status (positive vs. negative), or (2) SQ, adjusting for relevant covariates such as patient age and sex. A Firth penalized logistic regression was used to model the primary outcome, ICU mortality. Firth’s method was chosen to reduce small-sample bias and to handle potential issues of complete separation in the binary outcome data [24].

The LOS was analyzed using a generalized linear model with a Gamma distribution for the error term. A Gamma regression (with a log link) was selected because LOS is a non-negative, right-skewed variable, for which the Gamma distribution provides an appropriate modeling framework. The Gamma regression model included the same set of covariates as the logistic model, ensuring adjustment for baseline factors. To facilitate the interpretation of the regression results, we derived average marginal effects (AME) for each predictor. The AME represents the average change in the predicted probability of ICU mortality associated with a one-unit increase in a continuous predictor or with the presence of a categorical variable [25].

For the ICU mortality, the predictive ability of NECPAL and SQ was assessed by plotting the receiver operating characteristic (ROC) curve and calculating the area under the curve (AUC). An ROC AUC value closer to 1 indicates excellent discriminative ability of the model to distinguish between patients who did and did not experience the outcome.

A significance level of α = 0.05 was used for all statistical tests. Thus, *p*-values less than 0.05 were considered statistically significant. All confidence intervals presented are 95% confidence intervals (95% CI). For the regression models, effect estimates (such as odds ratios or marginal effects) are accompanied by 95% CIs to indicate the precision of the estimates. Statistical analyses were performed using R 3.4.2 [26].

### 2.7. Sample Size

The sample size was determined based on the primary objective of estimating the discriminatory performance of the NECPAL tool for ICU mortality, quantified by the area under the receiver operating characteristic (ROC) curve (AUC). We aimed to detect an AUC of 0.80, which represents good discriminatory ability, with a 95% confidence interval width not exceeding 0.10, and a two-sided alpha level of 0.05. Using the method described by Hanley and McNeil for estimating the standard error of the AUC [27], and assuming a mortality prevalence of 10%, a total sample size of 85 patients (including at least 8–9 events) was sufficient to achieve the desired precision.

## 3. Results

### 3.1. Baseline Characteristics

**Figure 1:**
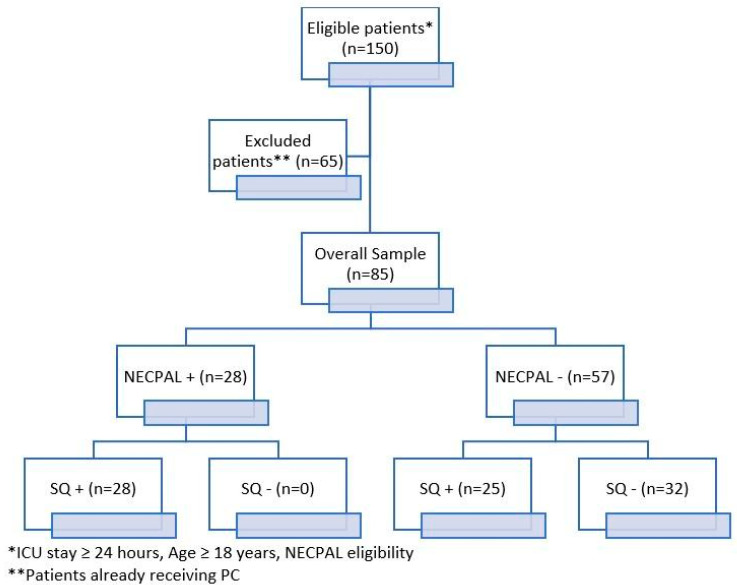
Study flowchart. NECPAL+ = positive test, NECPAL− = negative test; SQ+ = not surprised if patient dies; SQ− = surprised if pa-tient dies.

The characteristics at the baseline of the study population are reported in Table 1. A total of 85 critically ill adult patients were included in the study (Figure 1). No patients were excluded due to incomplete NECPAL data.

The median age was 73 [58–78] years, and 61.2% (*n* = 52) were males. Overall, 41.2% (*n* = 35) of patients were classified as frail, defined by a Clinical Frailty Scale (CFS) score ≥ 5, while 58.8% (*n* = 50) were considered non-frail. The median Karnofsky Performance Status was 70 [60–90], indicating a broad variability in functional baseline.

Most patients (82.4%, *n* = 70) experienced no more than one hospital admission in the 12 months preceding ICU admission, while 17.6% (*n* = 15) had been hospitalized two or more times. The most common reason for ICU admission was respiratory failure, accounting for 43.5% (*n* = 37) of cases, followed by infectious diseases (29.4%, *n* = 25) and cardiovascular conditions (17.6%, *n* = 15). Less frequent diagnoses included neurological (3.5%, *n* = 3), renal (1.2%, *n* = 1), and other causes (4.7%, *n* = 4). At the time of ICU admission, 68.2% (*n* = 58) of patients required invasive mechanical ventilation, 16.5% (*n* = 14) received non-invasive support, and 15.3% (*n* = 13) did not require any ventilatory assistance (Table 1).

### 3.2. Need for PC Assessed by NECPAL

Of the 85 patients enrolled, 28/85 (32.9%) were classified as NECPAL-positive and 57/85 (67.1%) as NECPAL-negative (Table 1).

NECPAL-positive patients were significantly older than NECPAL-negative ones, with a median age of 77 [73–80.2] vs. 66 [58–76] years (*p* = 0.001). Frailty was also more common in the NECPAL-positive group, where 64.2% (*n* = 18) had a CFS ≥ 5, compared to 29.8% (*n* = 17) among NECPAL-negative patients (*p* = 0.001).

The Karnofsky Performance Status (KPS) was lower in NECPAL-positive patients (55 [50–70] vs. 80 [70–90], *p* < 0.001). Comorbidities (≥2) were more frequent in NECPAL-positive patients (96.4%) than in NECPAL-negative patients (80.7%, *p* = 0.050). Admission origin differed between groups, with 17.9% of NECPAL-positive patients admitted from the emergency department, compared to 3.5% of NECPAL-negative patients (*p* = 0.028).

In terms of ICU admission diagnosis (*p* = 0.027), respiratory failure was more prevalent among NECPAL-negative patients (52.6% vs. 25.0%), while infectious (39.3% vs. 24.6%) and neurological causes (10.7% vs. 0%) were more frequent in NECPAL-positive patients. Cardiovascular diagnoses were similarly distributed between groups. No statistically significant differences were observed in pre-ICU length of stay [0.0 (0.0–4.5) vs. 1.0 (0.0–3.0) days, *p* = 0.415] or ventilatory support requirements. Invasive ventilation was the most common mode in both groups (64.3% in NECPAL-positive vs. 70.2% in NECPAL-negative, *p* = 0.829).

Indicators of functional and clinical vulnerability were more prevalent among NECPAL-positive patients. Specifically, dependence in activities of daily living was present in 75% of NECPAL-positive vs. 96.5% of NECPAL-negative patients; cognitive decline in 86% vs. 89.5%; and severe malnutrition in 96% vs. 100%, respectively. Although differences in some items were not statistically significant, this pattern reflects a higher burden of functional impairment and complexity among NECPAL-positive patients (Table S1).

### 3.3. NECPAL Items and Discrimination Capabilities

The Item 1 of the NECPAL, i.e., the SQ, was positive in all NECPAL-positive patients, whereas more than half of the NECPAL-negative patients did not meet this criterion. The differences among the other NECPAL items criteria are reported in Table S2. As concerns chronic conditions, nearly all NECPAL-positive patients met this condition, compared to none of the NECPAL-negative patients. Similarly, uncontrolled symptoms and frequent hospitalizations were more prevalent among NECPAL-positive patients, suggesting a greater healthcare burden and clinical complexity in this subgroup.

Functional impairment was another distinguishing factor, since NECPAL-positive patients were more likely to experience dependence in daily activities and cognitive decline, reflecting their overall higher level of frailty. While severe malnutrition was uncommon in both groups, persistent frailty was nearly universal among NECPAL-positive patients, further differentiating them from the NECPAL-negative group.

### 3.4. Outcome and NECPAL Status

NECPAL-positive patients had significantly higher ICU mortality rates (32.1%) compared to NECPAL-negative patients (1.8%, *p* < 0.001). The median ICU length of stay (ICU-LoS) was 11.0 [8.0–16.2] days in NECPAL-positive patients and 10.0 days [5,6,7,8,9,10,11,12,13,14] in NECPAL-negative patients (Table 2, Figure S1A). The length of ICU stay did not differ significantly between groups. NECPAL-positive patients had a median ICU stay of 11.0 [8.0–16.2] days, compared to 10.0 [5.0–14.0] days in NECPAL-negative patients (*p* = 0.364, Figure S1C).

### 3.5. Multivariable Models for ICU Mortality

**Figure 2:**
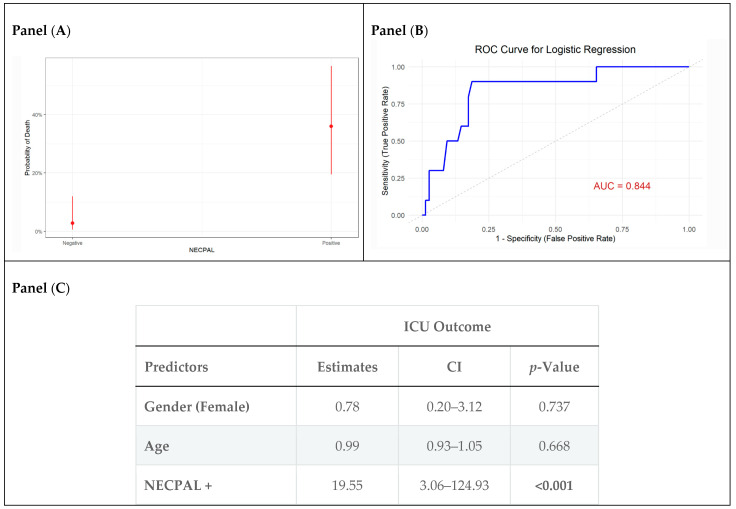
Firth penalized logistic regression model performance and estimates for ICU outcomes. Panel (**A**) illustrates the predicted probabilities of death based on NECPAL status. Panel (**B**) displays the ROC curve. Panel (**C**) summarizes the model estimates.

Multivariable logistic regression analysis demonstrated that NECPAL positivity was an independent predictor of ICU mortality, with an odds ratio (OR) of 19.55 (95% CI: 3.06–124.93, *p* < 0.001) (Figure 2C). The probability of ICU mortality is higher for NECPAL-positive patients (Figure 2A). Age and sex were not significant predictors of ICU mortality. The model demonstrated strong discriminative performance (AUC = 0.84), as indicated by the ROC curve in (Figure 2B).

When assessing the capability of the SQ to predict mortality, we observed a different magnitude of risk, as well as the predicted mortality probabilities for SQ-positive patients, that while elevated, do not reach the same levels as those seen in NECPAL-positive patients. Figure S2B displays the ROC curve for the SQ model, indicating its discriminative ability. Compared to NECPAL’s predictive power, the ROC curve for SQ also shows strong classification performance but with potentially less predictive ability, reaching an AUC of 0.77 (Figure S2B).

### 3.6. Multivariable Models for ICU Length of Stay

NECPAL positivity was not significantly associated with LOS after adjusting for covariates (Table S3, Panel A). However, controlling for covariates, in the multivariable Gamma regression model, SQ positivity was independently associated with an increased ICU LOS (AME: +5.6 days, 95% CI: 1.3–9.9, *p* = 0.01).

## 4. Discussion

In this study, we evaluated the prognostic utility of the NECPAL tool in identifying early PC needs among patients admitted to a general ICU. Our findings revealed that nearly one-third of ICU admissions during the study period required PC support as part of their overall treatment. Notably, the “SQ” yielded a positive response in all patients identified as NECPAL+, highlighting its effectiveness in screening for PC needs. Additionally, we assessed the tool’s ability to predict short-term outcomes, specifically ICU mortality and length of stay. A positive NECPAL classification was independently associated with increased ICU mortality, though no significant impact was observed on ICU length of stay.

These findings reinforce the value of early identification of PC needs as a complementary approach for patients, especially within the ICU, and not solely at the end-of-life stage [28]. The use of the NECPAL tool in this study has highlighted the significant PC needs of ICU patients, particularly those with chronic conditions [18]. This insight contributes to a more nuanced understanding of how palliative interventions can be integrated across various levels of care [29]. Importantly, a positive NECPAL result does not imply a one-size-fits-all approach to PC; instead, it underscores the importance of customizing interventions to address each patient’s specific needs.

While severity scores such as SOFA and APACHE are routinely used to predict short-term mortality in the ICU, their scope is limited to physiological derangements and acute organ dysfunction [30,31]. They do not incorporate indicators of chronic disease progression, functional decline, or psychosocial needs, which are central to identifying patients who may benefit from PC.

In our study, the NECPAL tool did not significantly predict ICU length of stay, whereas the SQ showed a modest association with ICU stay. This divergence is clinically meaningful. NECPAL integrates multidimensional indicators of chronic illness progression and palliative needs, which appear more strongly linked to the risk of ICU admission and mortality than to the dynamics of ICU stay once admitted.

Our findings suggest that while the SQ may serve as a rapid initial screening tool in the ICU, the NECPAL instrument provides a more accurate and multidimensional approach to risk stratification in critically ill patients. This distinction highlights the value of combining a quick bedside question with a structured tool that captures broader clinical and functional dimensions. By contrast, the SQ relies on clinician intuition, which may capture acute severity and fragility in the short term, factors more directly related to ICU resource utilization and length of stay. These findings suggest that different instruments provide complementary information: NECPAL may be better suited for identifying patients at risk of deterioration and future ICU admission, while the SQ may offer insight into likely ICU trajectories among those admitted. Clinically, this underscores the value of integrating structured prognostic tools with clinician judgment to support anticipatory care planning, family discussions, and resource allocation in the ICU.

Our findings add to existing ICU PC research by suggesting that NECPAL may complement severity scores, though its subjectivity and the single-center design limit generalizability; implementation in real-world settings will require clinician training, workflow integration, and collaboration between ICU and PC teams [32].

Such training enhances the reliability and consistency of PC assessments. Moreover, comprehensive and detailed medical documentation—particularly regarding subtle yet clinically relevant aspects of a patient’s condition—is essential for supporting a holistic evaluation. These steps are consistent with prior recommendations emphasizing the importance of education and accurate record-keeping in the effective deployment of prognostic tools like NECPAL.

This study has several limitations. First, the relatively small sample size restricts the generalizability of our findings, underscoring the need for larger multicenter studies to confirm external validity and to enable more robust adjustment for confounding, as well as more reliable subgroup and sensitivity analyses. Second, the NECPAL tool includes subjective components, particularly the SQ and item Q2_2, which may introduce variability in assessments; although this subjectivity is partly mitigated by its integration within NECPAL’s multidimensional framework, it remains a potential source of variability and using consensus among multiple professionals could improve reliability. Third, the exclusion of direct patient communication may have reduced the depth of assessment. Integrating these dimensions could improve its ability to capture quality-of-life concerns and care preferences, thereby enhancing its usefulness for guiding PC in the ICU. Lastly, while the NECPAL tool has a high negative predictive value, its lower specificity may lead to over-identification of at-risk patients [18,33]. Although this may encourage earlier integration of PC, it also presents challenges for appropriate resource allocation, primary PC recommendations for critical care clinicians [33].

Furthermore, as the NECPAL tool is primarily designed for patients with chronic illnesses, its use in the ICU should be complemented by tools that assess acute, life-threatening conditions. Discrepancies between NECPAL and SQ responses in this study underscore the need for additional instruments tailored to the complexities of critical illness, to better guide timely and appropriate end-of-life care.

## 5. Conclusions

This pilot study demonstrated that approximately one-third of ICU patients were identified by NECPAL as having PC needs, and these individuals exhibited a substantially higher risk of mortality. The integration of NECPAL with established ICU severity scores may improve the early recognition of PC needs and facilitate more informed, patient-centered decision-making. Future multicenter investigations with larger cohorts are warranted to validate these preliminary findings and to assess their implications for the quality of care in critical care settings.

## Figures and Tables

**Table 1:** Baseline characteristics of NECPAL+ and NECPAL− patients.

NECPAL		Overall	Positive	Negative	*p*-Value
Total, *n* (%)		85	28 (32.9)	57 (67.1)	
Males, *n* (%)		52 (61.2)	15 (53.6)	37 (64.9)	0.350
Age (years), Median (IQR)		73.0 (58.0 to 78.0)	77.0 (73.0 to 80.2)	66.0 (58.0 to 76.0)	0.001
Clinical Frailty Scale, *n* (%)	Frail (CFS ≥ 5)	35 (41.2)	18 (64.2)	17 (29.8)	0.001
	Non-frail (CFS < 5)	50 (58.8)	10 (35.8)	40 (70.2)	
Karnofsky Performance Status, Median (IQR)		70 (60.0 to 90.0)	55 (50.0 to 70.0)	80 (70.0 to 90.0)	<0.001
Number of hospital admissions in the 12 preceding months, *n* (%)	≤1	70 (82.4)	22 (78.6)	48 (84.2)	0.351
	≥2	15 (17.6)	6 (21.4)	9 (15.8)	
Comorbidities ≥ 2, *n* (%)		73 (85.9)	27 (96.4)	46 (80.7)	0.050
Wards before ICU admission, *n* (%)	Other ICU	3 (3.5)	0 (0.0)	3 (5.3)	0.028
	Floor	17 (20.0)	8 (28.6)	9 (15.8)	
	Operating room	58 (68.2)	15 (53.6)	43 (75.4)	
	Emergency department	7 (8.2)	5 (17.9)	2 (3.5)	
Pre-ICU admission LOS (days), Median (IQR)		1.0 (0.0 to 3.0)	0.0 (0.0 to 4.5)	1.0 (0.0 to 3.0)	0.415
ICU admission diagnosis, *n* (%)	Cardiovascular	15 (17.6)	6 (21.4)	9 (15.8)	0.027
	Infectious	25 (29.4)	11 (39.3)	14 (24.6)	
	Respiratory	37 (43.5)	7 (25.0)	30 (52.6)	
	Renal	1 (1.2)	0 (0.0)	1 (1.8)	
	Neurological	3 (3.5)	3 (10.7)	0 (0.0)	
	Others	4 (4.7)	1 (3.6)	3 (5.3)	
Ventilatory support, *n* (%)	Invasive	58 (68.2)	18 (64.3)	40 (70.2)	0.829
	Non-invasive	14 (16.5)	5 (17.9)	9 (15.8)	
	No support	13 (15.3)	5 (17.9)	8 (14.0)	

Definition of abbreviations: IQR = interquartile range; CFS = Clinical Frailty Scale; ICU = Intensive Care Unit; LOS = Length of Stay. Categorical variables were described using absolute frequencies (n) and percentages (%), while continuous variables were summarized using the median and interquartile range (IQR).

**Table 2:** Descriptive comparison for the outcomes between NECPAL+, NECPAL− (Panel A), SQ+, and SQ− patients (Panel B).

**Panel A NECPAL**				
		**NECPAL+**	**NECPAL−**	***p*-Value**
Total, n (%)		28 (32.9)	57 (67.1)	
ICU outcome, n (%)	Transferred	19 (67.9)	56 (98.2)	<0.001
	Dead	9 (32.1)	1 (1.8)	
ICU-LOS (days), Median (IQR)		11.0 (8.0 to 16.2)	10.0 (5.0 to 14.0)	0.364
**Panel B SQ**				
		**SQ+**	**SQ−**	***p*-Value**
Total, n (%)		53 (62.4)	32 (37.6)	
ICU outcome, n (%)	Transferred	43 (81.1)	32 (100.0)	0.011
	Dead	10 (18.9)	0 (0.0)	
ICU-LOS (days), Median (IQR)		11.0 (8.0 to 17.0)	8.5 (4.0 to 13.2)	0.098

Definition of abbreviations: IQR = interquartile range; ICU = Intensive Care Unit; LOS = Length of Stay. Categorical variables were described using absolute frequencies (n) and percentages (%), while continuous variables were summarized using the median and interquartile range (IQR).

## Data Availability

The datasets generated and/or analyzed during the current study are available from the corresponding author on reasonable request.

## Supplementary Materials

The following supporting information can be downloaded at https://www.mdpi.com/article/10.3390/jcm14176244/s1; Figure S1. Length of Stay (LoS) by NECPAL status, Surprise Question (SQ), and invasive strategy; Figure S2. Firth penalized logistic regression model performance and estimates for ICU outcomes; Table S1. Positivity rates for the different NECPAL items among the overall sample, NECPAL-positive, and NECPAL-negative patients; Table S2. Baseline characteristics of NECPAL+ and NECPAL− patients; Table S3. Multivariable Gamma model Average Marginal Effect (AME) estimates for NECPAL and SQ.
