# Supplementary material for: Early Detection of Palliative Care Needs in Critically Ill Patients Using the NECPAL Tool
Source: J Clin Med. 2025 Sep 4;14(17):6244. doi: 10.3390/jcm14176244 (PMC12429031; doi:10.3390/jcm14176244)
Supplement: Supplementary file 1 [file jcm-14-06244-s001.zip › jcm-3808353-supplementary.pdf]

# Supplement

| Variable | Overall Sample (N, %) | NECPAL Positive (N, %) | NECPAL Negative (N, %) |
|---|---|---|---|
| **Surprise Question (SQ) Response** | 85 | 28 (32.9) | 57 (67.1) |
| **Functional Decline (Q2_1)** | 81 (95.3) | 24 (85.7) | 57 (100.0) |
| **Chronic Condition Impact (Q2_2)** | 58 (68.2) | 1 (3.6) | 57 (100.0) |
| **Uncontrolled Symptoms (Q3_1)** | 79 (92.9) | 23 (82.1) | 56 (98.2) |
| **Frequent Hospitalizations (Q3_2)** | 76 (89.4) | 22 (78.6) | 54 (94.7) |
| **Severe Malnutrition (Q3_3)** | 84 (98.8) | 27 (96.4) | 57 (100.0) |
| **Cognitive Decline (Q3_4)** | 75 (88.2) | 24 (85.7) | 51 (89.5) |
| **Dependence in Activities of Daily Living (Q3_5)** | 76 (89.4) | 21 (75.0) | 55 (96.5) |
| **Persistent Frailty (Q3_6)** | 12 (14.1) | 1 (3.6) | 11 (19.3) |
| **Limited Social Support (Q4_1)** | 79 (92.9) | 22 (78.6) | 57 (100.0) |
| **Advanced Disease Progression (Q4_3)** | 82 (96.5) | 25 (89.3) | 57 (100.0) |
| **Recurrent Infections (Q4_4)** | 83 (97.6) | 26 (92.9) | 57 (100.0) |
| **End-of-Life Symptoms (Q4_5)** | 84 (98.8) | 27 (96.4) | 57 (100.0) |
| **Poor Prognosis Indicators (Q4_6)** | 84 (98.8) | 27 (96.4) | 57 (100.0) |

*Table S1: Positivity rates for the different NECPAL items among the overall sample, NECPAL-positive and NECPAL-negative patients. Definition of abbreviations: SQ=Surprise Question.*

| SQ | | Positive | Negative | P value |
|---|---|---|---|---|
| **Total n (%)** | | 53 (62.4) | 32 (37.6) | |
| **Males, n (%)** | | 30 (56.6) | 22 (68.8) | 0.359 |
| **Age (years), Median (IQR)** | | 77.0 (73.0 to 80.0) | 58.0 (51.5 to 64.0) | <0.001 |
| **Clinical Frailty Scale, n (%)** | Frail (CFS≥5) | 32 (60.4) | 3 (9.4) | <0.001 |
| | Non-frail (CFS≤4) | 21 (39.6) | 29 (90.6) | |
| **Karnofsky Performance Status, Median (IQR)** | | 60.0 (50.0 to 70.0) | 90.0 (80.0 to 90.0) | <0.001 |
| **Number of hospital admissions in the 12 preceding months, n (%)** | ≤1 | 43 (81.1) | 27 (84.4) | 0.702 |
| | ≥2 | 10 (18.9) | 5 (15.6) | |
| **Comorbidities ≥2, n (%)** | | 48 (90.6) | 25 (78.1) | 0.110 |
| **Origin before ICU admission, n (%)** | Other ICU | 0 (0.0) | 3 (9.4) | 0.075 |
| | Floor | 12 (22.6) | 5 (15.6) | |
| | Operating room | 35 (66.0) | 23 (71.9) | |
| | Emergency department | 6 (11.3) | 1 (3.1) | |
| **pre-ICU admission LOS (days), Median (IQR)** | | 0.0 (0.0 to 4.0) | 1.0 (0.0 to 3.0) | 0.164 |
| **ICU admission diagnosis, n (%)** | Cardiovascular | 11 (20.8) | 15 (17.6) | 0.414 |
| | Infectious | 16 (30.2) | 25 (29.4) | |
| | Respiratory | 20 (37.7) | 37 (43.5) | |

| | | | | |
|---|---|---|---|---|
| | Renal | 0 (0.0) | 1 (1.2) | |
| | Neurological | 3 (5.7) | 3 (3.5) | |
| | Others | 3 (5.7) | 4 (4.7) | |
| **Ventilatory support, n (%)** | Invasive | 38 (71.7) | 20 (62.5) | 0.662 |
| | Non-invasive | 8 (15.1) | 6 (18.8) | |
| | No support | 7 (13.2) | 13 (15.3) | |

*Table S2: Baseline characteristics of NECPAL + and NECPAL – patients. Definition of abbreviations: IQR=inter quartile range; CFS=Clinical Frailty Scale; ICU=Intensive Care Unit; LOS=Length of Stay. * p Values: obtained from comparative analysis among the two groups described: NECPAL + and NECPAL -. Categorical variables were described using absolute frequencies (n) and percentages (%), while continuous variables were summarized using the median and interquartile range (IQR).*

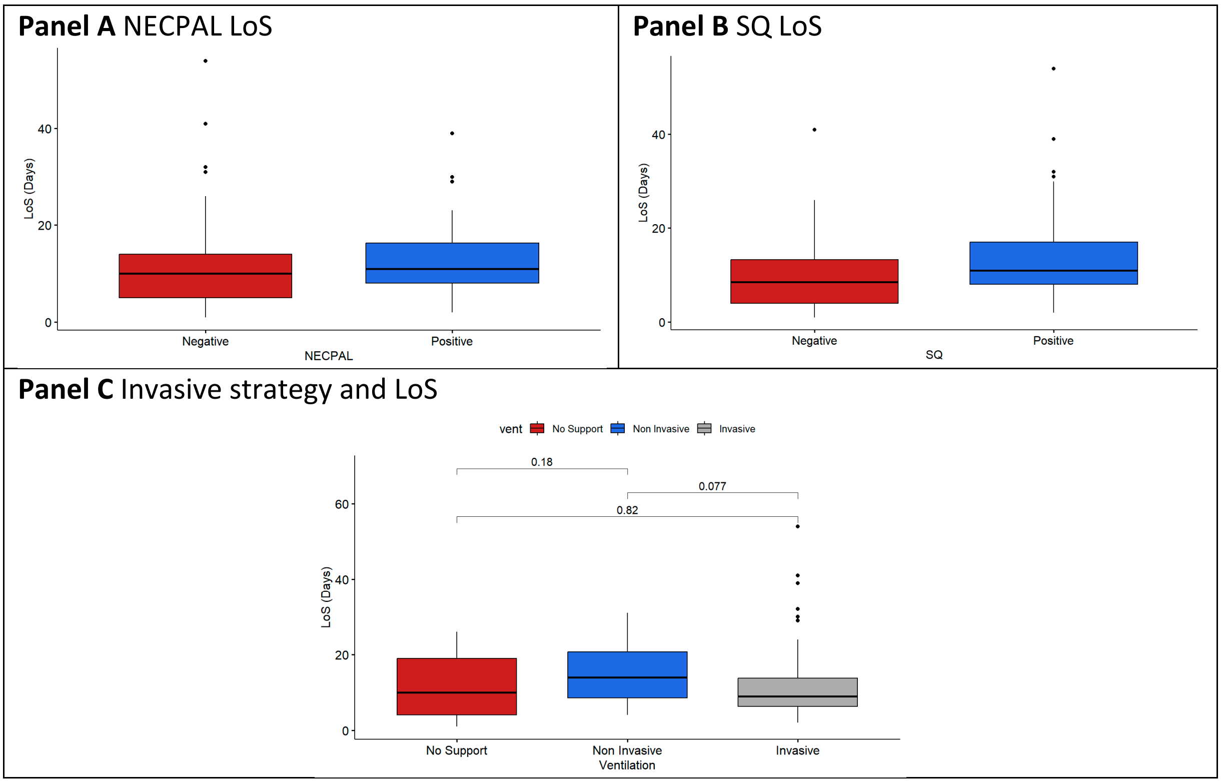


*Figure S1: Length of Stay (LoS) about NECPAL Status, Surprise Question (SQ), and Invasive Strategy*

- *Panel A: Comparison of Length of Stay (LoS) between patients classified as NECPAL positive and negative.*
- *Panel B: LoS distribution based on the Surprise Question (SQ) classification.*
- *Panel C: LoS across different levels of ventilatory support (No Support, Non-Invasive, Invasive), with p-values indicating statistical comparisons.*

**Panel A** SQ Model Prediction Plot

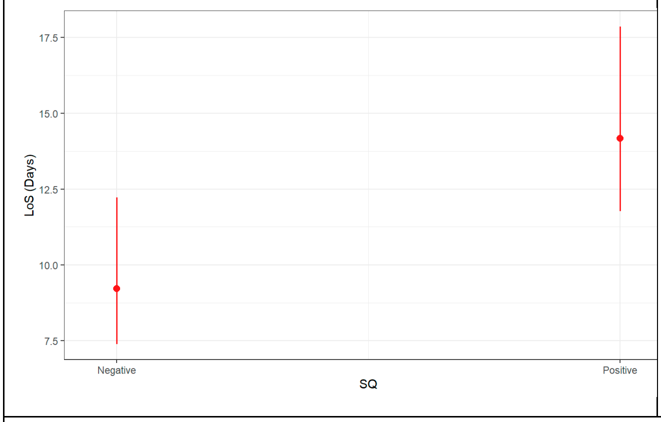


**Panel B** ROC Curve

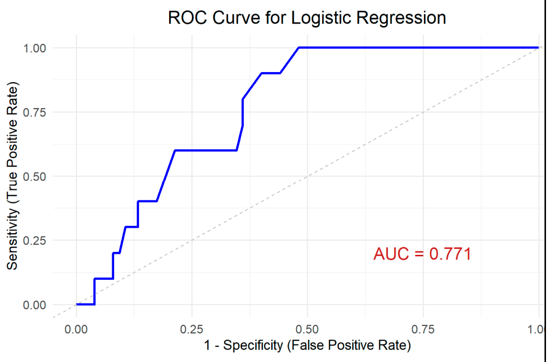


**Panel C** Model Estimates

| | **ICU outcome** | | |
|---|---|---|---|
| ***Predictors*** | *Odds Ratios* | *CI* | *P Value* |
| **Sex (Female)** | 0.9 | 0.24 – 3.37 | 0.881 |
| **Age** | 0.99 | 0.94 – 1.04 | 0.682 |
| **SQ (Positive)** | 18.35 | 1.06 – 317.41 | 0.009 |

*Figure S2: Firth Penalized Logistic Regression Model Performance and Estimates for ICU Outcomes. Panel A illustrates the predicted probabilities of death based on SQ (Surprise Question) status. Panel B displays the ROC curve. Panel C summarizes the model estimates.*

**Panel A** LoS Model NECPAL

| *Predictor* | *AME* | *95% CI Lower* | *95% CI Upper* | *P value* |
|---|---|---|---|---|
| **Sex (Female)** | -2.017 | -6.252 | 2.218 | 0.350 |
| **Age** | -0.118 | -0.278 | 0.042 | 0.149 |
| **NECPAL (Positive)** | 3.111 | -3.102 | 9.324 | 0.326 |

**Panel B** LoS Model SQ

| *Predictor* | *AME* | *95% CI Lower* | *95% CI Upper* | *P value* |
|---|---|---|---|---|
| **Sex (Female)** | -1.98 | -6.166 | 2.206 | 0.354 |
| **Age** | -0.150 | -0.299 | 0.000 | 0.05 |
| **SQ (Positive)** | 5.608 | 1.337 | 9.878 | 0.01 |

*Table S3 Multivariable Gamma model AME (Average Marginal Effect) estimates for NECPAL (Panel A) and SQ (Panel B).*
